# Exploring the use of machine learning for risk adjustment: A comparison of standard and penalized linear regression models in predicting health care costs in older adults

**DOI:** 10.1371/journal.pone.0213258

**Published:** 2019-03-06

**Authors:** Hong J. Kan, Hadi Kharrazi, Hsien-Yen Chang, Dave Bodycombe, Klaus Lemke, Jonathan P. Weiner

**Affiliations:** Center for Population Health IT, Department of Health Policy and Management, Johns Hopkins Bloomberg School of Public Health, Baltimore, Maryland, United States of America; University of Maribor, SLOVENIA

## Abstract

**Background:**

Payers and providers still primarily use ordinary least squares (OLS) to estimate expected economic and clinical outcomes for risk adjustment purposes. Penalized linear regression represents a practical and incremental step forward that provides transparency and interpretability within the familiar regression framework. This study conducted an in-depth comparison of prediction performance of standard and penalized linear regression in predicting future health care costs in older adults.

**Methods and findings:**

This retrospective cohort study included 81,106 Medicare Advantage patients with 5 years of continuous medical and pharmacy insurance from 2009 to 2013. Total health care costs in 2013 were predicted with comorbidity indicators from 2009 to 2012. Using 2012 predictors only, OLS performed poorly (e.g., R^2^ = 16.3%) compared to penalized linear regression models (R^2^ ranging from 16.8 to 16.9%); using 2009–2012 predictors, the gap in prediction performance increased (R^2^:15.0% versus 18.0–18.2%). OLS with a reduced set of predictors selected by lasso showed improved performance (R^2^ = 16.6% with 2012 predictors, 17.4% with 2009–2012 predictors) relative to OLS without variable selection but still lagged behind the prediction performance of penalized regression. Lasso regression consistently generated prediction ratios closer to 1 across different levels of predicted risk compared to other models.

**Conclusions:**

This study demonstrated the advantages of using transparent and easy-to-interpret penalized linear regression for predicting future health care costs in older adults relative to standard linear regression. Penalized regression showed better performance than OLS in predicting health care costs. Applying penalized regression to longitudinal data increased prediction accuracy. Lasso regression in particular showed superior prediction ratios across low and high levels of predicted risk. Health care insurers, providers and policy makers may benefit from adopting penalized regression such as lasso regression for cost prediction to improve risk adjustment and population health management and thus better address the underlying needs and risk of the populations they serve.

## Introduction

Risk adjustment models are applied by payers and health care delivery organizations to adjust for differences in patient characteristics when estimating expected health care resource use, clinical outcomes, and quality of care. Commonly used predictors in risk adjustment models include demographic information and clinical variables. The dominant type of risk adjustment models in practice are standard linear regression based on ordinary least squares (OLS) [1]. For example, the HHS-Hierarchical Condition Categories (HHS-HCC) model, a risk adjustment model adopted for health plans participating in the Affordable Care Act, uses standard linear regression with age, gender, diagnoses and interactions between diagnoses to predict medical expenditure risk [2].

An emerging literature has begun to explore the potential application of machine learning methods to predict health care costs and utilization for risk adjustment purposes [3–6]. These studies compared a variety of machine learning techniques for risk adjustment including penalized regression, random forests, multivariate adaptive regression splines, boosted regression trees, neural network, and super learner. Early success has demonstrated the potential value of machine learning regression and classification methods for predicting costs and utilization. With new data sources becoming available for population health management [7–9], machine learning methods will become increasingly useful to process and analyze increasingly complex population-level health data.

However, despite the potential value of advanced machine learning approaches to predicting risk, payers and providers are still heavily relying on OLS regression to risk adjust and manage their patient populations. The slow adoption of advanced machine learning techniques can be partly explained by the unfamiliarity of risk stratification analysts with such techniques and complex interpretation and integration of results needed in practice. One approach to pushing the needle toward machine learning adoption in risk adjustment practice is through the introduction of incremental, effective and transparent machine learning regression models that stay within the framework of standard linear regression and also have as good performance as some more sophisticated but less transparent machine learning techniques [3]. This study concentrated on penalized linear regression models including lasso (least absolute shrinkage and selection operator) [10], ridge [11] and elastic net [12] and conducted a thorough comparison of penalized regression with standard linear regression in predicting total health care costs, which was not previously reported in published literature. We focused on older adults (≥65 years old) as they incur disproportionately more health care spending [13].

Multiple factors make penalized linear regression a viable potential next step beyond OLS for risk prediction and adjustment. First, transparency of a risk adjustment model is paramount for care management and resource allocation. Penalized linear regression provides almost the same level of transparency and interpretability as standard linear regression. Some machine learning techniques such as random forests and neural network are hard to estimate and difficult to interpret, and yet they do not offer better prediction compared to penalized regression in predicting health care costs [3]. Second, despite that standard linear regression is still the most popular risk adjustment approach, penalized linear regression can be as easily scaled and deployed in environments with limited computational power and thus represents a pragmatic step forward for risk adjustment. Third, penalized regression such as lasso regression selects and retains important variables for prediction. Providers often have incentives to increase the intensity of coding medical services (a practice referred to as “upcoding”), especially those included in a risk adjustment model, in order to maximize reimbursement [14]. Carefully selecting predictors for a risk adjustment model with clinical insights and statistical criteria may curtail the opportunity for upcoding. As an example, HCC models accomplished this by creating a hierarchy of grouped conditions only based on a subset of all available diagnosis codes [2]. In addition, keeping only important variables in a model may facilitate care management as it is easier for care managers to target key risk factors.

The study also assessed the value of penalized regression in generating more parsimonious models as well as using additional predictors collected over a longer period of time. We tested parsimonious OLS models by including only important predictors selected by lasso regression. OLS provides unbiased estimates when specified correctly whereas penalized regression sacrifices unbiasedness for a potential reduction of expected prediction error. Variable selection may reduce the number of irrelevant predictors included in a model and thus increase efficiency and reduce the chance of overfitting. We also compared predictive model performance using baseline predictors from 1 year versus 4 years in the past.

The overall goal of this study was to assess the potential of penalized linear regression models for risk adjustment. Specifically, the study 1) compared standard linear regression with penalized linear regression in predicting future total health care costs in older adults, 2) compared standard linear regression using full and reduced sets of predictors selected by lasso regression, and 3) assessed the value of using longitudinal data from 4 years versus 1 year in the past as predictors.

## Methods

This retrospective cohort study used IMS LifeLink Health Plan Claims Database [15], which is comprised of fully adjudicated and de-identified medical and pharmaceutical claims from health insurance plans. The database captures a geographically diverse sample of health plan enrollees in the U.S. Charges, allowed and paid amounts are available for all services rendered, as well as date of service for all claims. The database is fully compliant with the Health Insurance Portability and Accountability Act (HIPAA). The Institutional Review Board at the Johns Hopkins Bloomberg School of Public Health reviewed the study proposal and determined that the human subjects research activity described in the application meets the criteria for Exemption under 45 CFR 46.101(b), Category (4). It approved proposed use of an existing limited data set from commercial health plan claims in the U.S. (IRB No: 00008699). Patients were selected from a large health plan with longitudinal patient records. Patients were required to have 5 years of continuous medical and pharmacy insurance benefits from 2009 to 2013 and be at least 65 years old at the end of 2012. Although they were all Medicare Advantage enrollees, the selected patients were not nationally representative of Medicare Advantage enrollees.

Total health care costs in 2013 were the target outcome for all predictive models. Predictors were extracted from data prior to 2013. Previous diseases and symptoms as indicated by recorded medical diagnoses and pharmacy claims were included as predictors. The Johns Hopkins Adjusted Clinical Groups (ACG) System version 11.0 [16] was applied to medical and pharmacy claims to generate binary comorbidity indicators by grouping International Classification of Diseases, Ninth Revision, Clinical Modification (ICD-9-CM) diagnosis codes from inpatient and outpatient claims and National Drug Codes (NDCs) from pharmacy claims. Only diagnoses made by a physician (excluding labs, imaging, and other provisional diagnoses) were included for grouping. The high-level “rolled-up” comorbidity groups have up to 282 diagnosis-based conditions called Expanded Diagnosis Clusters (EDCs) and up to 67 pharmacy-based conditions called Rx-defined Morbidity Groups (RxMGs). EDC and RxMG grouping algorithms were created by clinicians based on clinical judgement and cover a large aggregate set of comorbidities. RxMGs represent conditions treated with medications and do not completely overlap with EDCs which are based solely on diagnosis codes. Comorbidities with zero prevalence were excluded. In addition to the yearly comorbidity indicators, age (at the end of 2012), age squared and sex were included as predictors in all predictive models. To compare predictive model performance using information from baseline periods of different length, yearly EDC and RxMG comorbidity indicators were first extracted from medical and pharmacy claims in 2012 for 1-year prospective prediction models, and then 4 sets of the same yearly indicators were extracted in each of the 4 years from 2009 to 2012 for longitudinal prediction models.

The primary difference between standard and penalized regression is that penalized regression adds a regularization term in a least squares loss function before it is optimized to estimate coefficients. Lasso regression adds the sum of absolute values of coefficient estimates as the regularization term (i.e., L1 regularization) whereas ridge regression adds the sum of squares of coefficient estimates as the regularization term (i.e., L2 regularization). Elastic net adds a weighted average of L1 and L2. One unique feature of lasso regression is that it selects predictors simultaneously with model estimation. We compared standard linear regression with penalized linear regression with lasso (α = 1), ridge (α = 0), and elastic net (0<α<1) regularization as defined by (1- α)/2ǁβǁ_2_^2^+ αǁβǁ_1_ (β is a vector of coefficients). We tested elastic net regularization with α ranging from 0.1 to 0.9 with an interval of 0.1. The regularization term is multiplied by a model hyperparameter called lambda that determines the total amount of regularization when added to the least squares loss function. This study used cross-validation to find the optimal value of lambda that achieved minimum cross-validation mean standard error [17]. In addition, we tested two parsimonious OLS models with 2012 and 2009–2012 predictors, including only predictors selected by lasso regression. The OLS regression predicting 2013 costs with the full set of 2012 predictors represented the standard base case model for comparison purposes.

The entire study sample was split into training (75%) and test (25%) sets. All model development and validation was conducted in the training set. OLS was estimated in the training set directly as no model tuning is needed. Penalized linear regression was tuned using 10-fold cross-validation in the training set. Tuned penalized regression models were re-estimated using the entire training dataset. Predictive performance of final estimated models was assessed in the test set by: (1) R squared (R^2^), representing the percent of total variation of actual costs explained by a model (a higher percent indicates better performance), (2) root mean squared error (RMSE): square root of mean squared differences between predicted and actual costs (a smaller value indicates better performance), (3) mean absolute prediction error (MAPE): mean absolute value of differences between predicted and actual costs (a smaller value indicates better performance), and (4) prediction ratio (PR): sum of predicted costs divided by sum of actual costs (a value closer to 1 indicates better performance). Model performance was assessed in the entire test set as well as within each of the 10 deciles of predicted costs in the test set. All programming was performed in R version 3.4.2 [18] with glmnet version 2.0–16 [19]. R codes can be found at https://github.com/hkan2018/risk_adjustment_with_penalized_regression.

## Results

A total of 81,106 patients met the selection criteria with 60,737 split to a training set and 20,369 to a test set. In the entire study sample, mean (standard deviation (SD)) age was 73.8 (6.7) years old and 50.8% were females. Mean total health care costs (SD) in 2013 was $16,509 (41,376). Proportion of patients with a specific EDC (n = 277) or RxMG (n = 67) in 2012 in the training set can be found in S1 Table.

Table 1 shows the performance of all the predictive models using 2012 predictors assessed in the test set. The OLS model with the full set of 2012 predictors assessed in the training set had an R^2^ of 18.5% (data not included in the table) versus 16.3% assessed in the test set, indicating some overfitting. OLS performed poorly, based on R^2^ (16.3%), RMSE (35,801) and MAPE (15,331), compared to ridge, elastic net and lasso penalized regression models, all of which displayed similar performance with R^2^ ranging from 16.8 to 16.9%, RMSE 35,669–35,690, and MAPE 15,244–15,260. However, the prediction ratio of OLS in the entire test set was 1.001 (note that this ratio assessed in the training set would be exactly 1 by the nature of standard linear regression), compared to the prediction ratios of penalized regression models (1.002–1.003), indicating a minor increase in bias of estimates of penalized linear regression as measured by prediction ratio.

**Table 1:** Prediction performance of models using 2012 predictors in predicting 2013 costs in the test set (n = 20,369).

	Mean predicted costs ($)	Mean actual costs ($)	R^2^	RMSE ($)	MAPE ($)	PR
OLS with all 2012 predictors	16,299	16,284	16.3%	35,801	15,331	1.001
OLS with lasso selected variables	16,307	16,284	16.6%	35,749	15,237	1.001
Ridge regression	16,320	16,284	16.9%	35,680	15,260	1.002
Elastic net regression						
0.1	16,337	16,284	16.9%	35,669	15,244	1.003
0.2	16,337	16,284	16.9%	35,679	15,250	1.003
0.3	16,337	16,284	16.9%	35,683	15,249	1.003
0.4	16,336	16,284	16.9%	35,686	15,249	1.003
0.5	16,336	16,284	16.9%	35,687	15,249	1.003
0.6	16,336	16,284	16.8%	35,688	15,249	1.003
0.7	16,336	16,284	16.8%	35,689	15,249	1.003
0.8	16,336	16,284	16.8%	35,689	15,249	1.003
0.9	16,336	16,284	16.8%	35,690	15,249	1.003
Lasso regression	16,336	16,284	16.8%	35,690	15,249	1.003

OLS: ordinary least squares; RMSE: root mean squared error; MAPE: mean absolute prediction error; PR: prediction ratio; lasso: least absolute shrinkage and selection operator

Out of the 347 original predictors, lasso regression selected 175 important variables including age and sex with coefficient estimates of all the other predictors shrunk to zero. Using these 175 variables, OLS performance improved (R^2^ = 16.6%, RMSE = 35,749, MAPE = 15,237) relative to OLS with the full set of 347 predictors (R^2^ = 16.3%, RMSE = 35,801, MAPE = 15,331). However, the performance of the parsimonious OLS model still lagged behind those of penalized regression models based on R^2^ (16.6% versus 16.8–16.9%) and RMSE (35,749 versus 35,669–35,690), although the MAPE measure for the parsimonious model showed a small improvement (15,237 versus 15,244–15,260). In addition, the parsimonious OLS model retained the same smaller prediction ratio (1.001) as the OLS model with the full set of predictors. See S1 Table for top 50 most prevalent 2012 EDC and RxMG comorbidity indicators selected by the lasso model.

Table 2 shows model performance within each of the 10 deciles of predicted costs for OLS (with the full and reduced sets of predictors), ridge, and lasso regression using 2012 predictors. Among the 4 models, lasso regression showed prediction ratios consistently close to 1 across all the 10 deciles of predicted costs (e.g., PR of 0.979 in decile 1 and 1.019 in decile 10). OLS with the full set of predictors under-predicted costs in low predicted risk deciles and over-predicted costs in high predicted risk deciles (e.g., PR of .433 in decile 1 and 1.073 in decile 10). Although the parsimonious OLS and ridge regression improved on prediction ratio compared to OLS with the full set of predictors, both the models showed inferior prediction ratios in low and high ends of predicted costs compared to lasso regression (e.g., PR of .539 in the parsimonious OLS and 0.754 in ridge regression compared to .979 in lasso regression in decile 1; PR of 1.075 in the parsimonious OLS and 1.022 in ridge regression compared to 1.019 in lasso regression in decile 10). Elastic net regression showed similar performance by deciles as lasso regression (see Table A in S2 Table).

**Table 2:** Prediction performance of models using 2012 predictors in predicting 2013 costs in the test set, by deciles of predicted costs.

Decile	N	Mean predicted costs ($)	Mean actual costs ($)	RMSE ($)	MAPE ($)	PR	Mean predicted costs ($)	Mean actual costs ($)	RMSE ($)	MAPE ($)	PR
		OLS with all 2012 predictors					OLS with lasso selected predictors				
1	2,037	1,998	4,616	19,838	5,059	0.433	2,104	3,905	16,269	4,544	0.539
2	2,037	4,343	5,121	16,931	5,768	0.848	4,420	4,889	18,801	5,618	0.904
3	2,037	6,288	6,826	17,929	7,205	0.921	6,395	7,768	23,361	7,821	0.823
4	2,037	8,415	8,945	23,073	8,686	0.941	8,492	8,530	18,981	8,400	0.996
5	2,037	10,703	11,527	24,208	11,090	0.928	10,766	11,550	24,658	11,113	0.932
6	2,037	13,336	14,100	28,423	13,523	0.946	13,396	13,636	25,046	12,955	0.982
7	2,037	16,530	17,403	30,072	16,208	0.950	16,536	17,979	32,054	16,577	0.920
8	2,037	20,843	19,854	33,412	18,371	1.050	20,788	19,325	30,683	17,695	1.076
9	2,037	27,689	25,201	37,021	22,821	1.099	27,618	26,374	40,338	23,550	1.047
10	2,036	52,867	49,260	80,627	44,593	1.073	52,576	48,898	80,165	44,117	1.075
		Ridge regression					Lasso regression				
1	2,037	2,750	3,648	15,132	4,505	0.754	3,504	3,579	15,115	4,894	0.979
2	2,037	4,844	6,045	23,024	6,852	0.801	5,439	5,097	18,625	6,338	1.067
3	2,037	6,724	6,298	13,349	6,823	1.068	7,202	6,987	17,470	7,521	1.031
4	2,037	8,778	9,244	23,858	9,144	0.950	9,083	8,939	23,624	8,992	1.016
5	2,037	10,967	11,545	23,703	11,137	0.950	11,125	11,666	23,490	11,205	0.954
6	2,037	13,544	13,719	27,210	13,296	0.987	13,566	14,144	28,889	13,652	0.959
7	2,037	16,625	17,426	30,711	16,126	0.954	16,525	17,439	31,803	16,352	0.948
8	2,037	20,799	19,520	30,661	17,895	1.066	20,395	19,547	29,879	17,442	1.043
9	2,037	27,419	25,741	39,450	23,129	1.065	26,640	26,482	39,961	23,066	1.006
10	2,036	50,772	49,667	80,528	43,708	1.022	49,893	48,971	80,089	43,036	1.019

OLS: ordinary least squares; RMSE: root mean squared error; MAPE: mean absolute prediction error; PR: prediction ratio; lasso: least absolute shrinkage and selection operator

The longitudinal predictive model included 1,387 predictors over the 4-year period from 2009 to 2012. Table 3 shows the same direction of performance gaps between standard and penalized linear regression with 4 years of predictors as shown by the models with 1 year of data, but the performance gaps enlarged as indicated by R^2^, RMSE and MAPE. For example, the difference in R^2^ between OLS with the full set of predictors (15.0%) and penalized regression models with 4 years of predictors (18.0–18.2%) was larger than between the models with 1 year of data (16.3% versus 16.8–16.9%). However, penalized regression with 4 years of data showed a slightly larger prediction ratio (1.004) compared to 1.002–1.003 in penalized regression with 1 year of data.

**Table 3:** Prediction performance of models using 2009–2012 predictors in predicting 2013 in the test set (n = 20,369).

	mean predicted costs	mean actual costs	R^2^	RMSE	MAPE	PR
OLS with all 2009–2012 Predictors	16,299	16,284	15.0%	36,077	16,111	1.001
OLS with lasso selected variables	16,298	16,284	17.4%	35,563	15,307	1.001
ridge regression	16,347	16,284	18.0%	35,448	15,279	1.004
elastic net regression						
0.1	16,351	16,284	18.2%	35,402	15,208	1.004
0.2	16,348	16,284	18.1%	35,419	15,208	1.004
0.3	16,347	16,284	18.1%	35,427	15,207	1.004
0.4	16,347	16,284	18.0%	35,431	15,207	1.004
0.5	16,347	16,284	18.0%	35,434	15,207	1.004
0.6	16,347	16,284	18.0%	35,435	15,207	1.004
0.7	16,346	16,284	18.0%	35,437	15,207	1.004
0.8	16,346	16,284	18.0%	35,438	15,207	1.004
0.9	16,346	16,284	18.0%	35,438	15,207	1.004
Lasso regression	16,346	16,284	18.0%	35,439	15,207	1.004

OLS: ordinary least squares; RMSE: root mean squared error; MAPE: mean absolute prediction error; PR: prediction ratio; lasso: least absolute shrinkage and selection operator

Improved performance of penalized regression models with 4 years versus 1 year of predictors (R^2^: 18.0–18.2% versus16.8–16.9%) indicates the value of longitudinal data for better prediction performance. However, this gain only occurred with penalized regression. OLS with full 2009–2012 predictors actually had worse performance (e.g., R^2^ = 15.0%) than OLS with full 2012 predictors (R^2^ = 16.3%). It is noteworthy that R^2^ of OLS with 2009–2012 predictors assessed in the training set was 21.5% vs. 15.0% in the test set, indicating more serious overfitting. However, OLS with important predictors over 4 years selected by lasso performed better (e.g., R^2^ = 17.4%) than OLS with full 2012 predictors (R^2^ = 16.3%).

Out of the original 1,387 predictors over the 4-year period, lasso regression selected 276 important predictors, among which 46, 44, 65 and 119 comorbidity indicators came from 2009, 2010, 2011, and 2012, respectively, indicating that all of the 4 previous years of data contributed to prediction of 2013 health care costs with more recent years of comorbidities more likely being selected as important variables. Although the parsimonious OLS regression (e.g., R^2^ = 17.4%) performed better than OLS with the full set of 2009–2012 variables (R^2^ = 15.0%), it still fell short of the performance achieved by penalized regression (R^2^: 18.0–18.2%), indicating that variable selection for OLS was not enough to achieve the same level of prediction improvement displayed by penalized regression.

Table 4 shows model performance by deciles of predicted costs with 4 years of predictors of the same 4 models (i.e., OLS with full and reduced sets of 2009–2012 predictors, ridge, and lasso regression). Comparing Table 2 and Table 4 shows more pronounced differences in prediction ratios between lasso and the other three models with 4 years of predictors. Prediction ratios of lasso regression were much closer to 1 across low and high levels of predicted costs compared to the other three models (e.g., PR of -0.177 in OLS with the full set of predictors, 0.316 in the parsimonious OLS, and 0.611 in ridge regression, compared to 1.106 in lasso regression in decile 1; PR of 1.164 in OLS with the full set of predictors, 1.109 in the parsimonious OLS, and 1.005 in ridge regression, compared to 1.004 in lasso regression in decile 10). Elastic net regression showed similar performance by deciles as lasso regression (see Table B in S2 Table).

**Table 4:** Prediction performance of models using 2009–2012 predictors in predicting 2013 costs in the test set, by deciles of predicted costs.

Decile	n	Mean predicted costs ($)	Mean actual costs ($)	RMSE ($)	MAPE ($)	PR	Mean predicted costs ($)	Mean actual costs ($)	RMSE ($)	MAPE ($)	PR
		OLS with all 2009–2012 Predictors					OLS with lasso selected predictors				
1	2,037	-1,287	7,272	26,095	8,893	-0.177	1,237	3,917	14,587	4,367	0.316
2	2,037	3,028	4,837	15,373	4,968	0.626	3,745	5,420	21,244	5,594	0.691
3	2,037	5,254	7,973	23,957	7,898	0.659	5,858	7,645	23,731	7,459	0.766
4	2,037	7,666	8,913	20,208	8,693	0.860	7,992	9,594	22,797	9,142	0.833
5	2,037	10,317	10,886	20,023	10,769	0.948	10,407	10,919	21,154	10,371	0.953
6	2,037	13,321	15,062	33,175	14,740	0.884	13,257	14,564	28,606	13,692	0.910
7	2,037	17,065	16,322	29,289	15,840	1.046	16,610	16,510	28,610	15,636	1.006
8	2,037	21,919	18,787	30,680	18,561	1.167	21,223	19,620	33,482	18,520	1.082
9	2,037	29,665	24,644	37,632	24,453	1.204	28,542	25,854	37,516	23,327	1.104
10	2,036	56,062	48,156	80,008	46,308	1.164	54,126	48,809	79,309	44,979	1.109
		Ridge regression					Lasso regression				
1	2,037	2,240	3,665	12,741	4,215	0.611	3,495	3,161	12,718	4,496	1.106
2	2,037	4,539	5,915	23,495	6,594	0.767	5,405	5,414	19,284	6,429	0.998
3	2,037	6,588	6,757	18,959	7,124	0.975	7,176	7,123	21,430	7,607	1.007
4	2,037	8,745	9,346	21,572	9,278	0.936	9,066	9,359	21,522	9,478	0.969
5	2,037	11,037	11,671	25,112	11,403	0.946	11,175	11,106	23,795	10,759	1.006
6	2,037	13,735	13,693	25,770	13,591	1.003	13,597	14,533	28,503	13,829	0.936
7	2,037	16,956	17,188	31,462	16,249	0.986	16,511	16,988	30,171	15,938	0.972
8	2,037	21,291	19,022	30,039	17,920	1.119	20,516	19,904	33,420	17,862	1.031
9	2,037	28,218	25,718	38,088	23,113	1.097	26,748	25,654	37,234	22,586	1.043
10	2,036	50,141	49,877	79,946	43,321	1.005	49,789	49,611	79,458	43,105	1.004

OLS: ordinary least squares; RMSE: root mean squared error; MAPE: mean absolute prediction error; PR: prediction ratio; lasso: least absolute shrinkage and selection operator

## Discussion

Payers and providers commonly use standard OLS linear regression for risk adjustment and population health management. Although machine learning methods in general have shown initial promising results, payers and providers have been slow in adopting unfamiliar complex methods with difficult-to-interpret results. However, they might be more amenable to techniques such as penalized linear regression with underlying machine learning fundamentals but familiar and transparent regression framework. This study demonstrated important advantages of using penalized regression versus traditional standard OLS regression to predict future healthcare costs among older adults with demographic and comorbidity variables.

Specifically, our findings showed that penalized linear regression outperformed OLS with full and reduced (selected by lasso) sets of predictors, based on R^2^, RMSE, and MAPE, except for prediction ratio in which OLS showed a slight advantage. Although all penalized regression models performed similarly when evaluated in the entire test set, lasso regression consistently showed superior prediction ratios across high and low levels of predicted risk compared to ridge and OLS. Coefficient shrinkage and variable selection may have helped lasso to achieve better performance across the entire risk spectrum. Built-in variable selection of lasso regression may reduce overfitting as well as the number of irrelevant predictors included in the model. In addition, lasso regression generated a much smaller number of negative predicted costs with only 2 observations in the test set with negative predictions compared to 120 negative predictions by the OLS model (data not shown). Although elastic net regression showed similar performance as lasso within deciles of predicted risk, lasso regression may be preferable for its simpler interpretation with built-in variable selection. In contrast, OLS suffers from biased prediction as indicated by prediction ratio deviating from 1 in low and high risk patients. Alleviating group-level biased prediction is critical to a health plan or a clinical care organization that may enroll a biased population of patients with underlying risk skewed towards either the high or low end of risk spectrum.

This study also demonstrated better prediction of parsimonious OLS models with a smaller set of important comorbidity indicators selected by lasso regression than OLS with the full set of predictors. OLS using a full set of predictors without any variable selection may suffer from including irrelevant predictors leading to increased standard error of estimates [20] and/or overfitting. In practice, including only important predictors in a risk adjustment model can both reduce opportunities for upcoding and facilitate care management by allowing care managers to focus on patients with key risk factors.

This study also compared predictive performance of OLS versus penalized regression models against various temporal cuts of the data to simulate situations where “longer” health care data is available (e.g., Medicare data). Comorbidities from each of the past 4 years contributed to better prediction by penalized regression compared to using only 1 year of prior data, and this gain in performance with longitudinal data can only be harnessed by penalized regression as standard linear regression actually showed worse performance using 4 years of prior predictors. We also compared overall performance of OLS and lasso regression with 1, 2, 3, and 4 years of prior data and saw a clean trend that with an increasing number of years of prior data, OLS lost prediction power while lasso gained prediction power (data not shown). This further confirms the advantage of using penalized regression such as lasso regression to model longitudinal data. Both payer and provider organizations can utilize this advantage of penalized regressions to increase the utility of their longer historical data that they are accumulating over time.

Although OLS may produce unbiased estimates when specified correctly, in practice, we do not expect a risk adjustment model for health care costs to be correctly specified, meaning incorporating only relevant variables and relating them to the cost outcome with correct functional specification. This is because individuals are exposed to numerous factors related to biology, behavior, health care, social and physical environment that may impact their health and health care through numerous complex and interactive pathways. Thus, it is not advisable to use causal inference and unbiased estimates to guide model selection for risk adjustment models. In this case, techniques like penalized regression that accept some bias in model estimates for a reduction in variance can be appropriate for improving overall expected prediction error. A favorable bias-variance tradeoff was clearly demonstrated for penalized regression in this study. Although penalized regression models produced slightly increased bias as measured by a 1% to 3% increase in prediction ratios relative to that of OLS in the entire test set, overall, penalized regression clearly achieved better prediction performance than OLS with and without variable selection. Furthermore, penalized regression, especially lasso and elastic net regression, even considerably improved on prediction ratios across low and high levels of predicted risk compared to OLS.

Numerous machine learning techniques exist for regression in the supervised learning setting [17]. Although some machine learning methods such as super learner [3] and deep learning [21] may boost prediction accuracy, they are usually not easy to train nor to understand and interpret, and may require substantial computing power. A transparent modeling technique such as lasso regression is easier to train and scale and empirically demonstrated superior performance among all the other standard and penalized linear regression models tested in this study.

This study only used comorbidity indicators as predictors, derived from recorded diagnoses and filled prescription drugs, reflecting the information a primary care physician (PCP) may typically have access to. A PCP usually knows relatively well diseases and symptoms as well as prescription drugs of his/her patients. Even without complete information, lasso regression demonstrated that only a subset of comorbidities was important for predicting costs. In addition, despite the lack of comprehensive information on health care costs and utilization in EHR systems [22,23], EHRs provide unique data sources for risk stratification [24–28]. Thus, the findings of this study are potentially applicable to both provider and payer settings for practical risk adjustment applications [29].

Finally, although this study did not intend to develop a full risk adjustment model ready to use for payment purposes, it is still worth noting that estimating the impact of improved risk adjustment on actual outcomes such as adverse selection and overpayments to health plans is not as straightforward as it may first appear to be because of the need to consider endogenous response of payers to specific incentives created by a risk adjustment model [30]. For example, an increase in R^2^ of a risk adjustment formula does not necessarily result in an increase or a decrease of government overpayments in the Medicare Advantage program [31]. An empirical investigation of the Hierarchical Condition Categories (HCCs) risk adjustment approach developed by the Centers for Medicare and Medicaid Services (CMS) found that the introduction of the more sophisticated risk adjustment did not alter favorable selection into Medicare Advantage [32]. But we expect that more accurate prediction of costs especially across different levels of risk as demonstrated by penalized regression such as lasso may reduce the room for possible adverse selection and thus make it more difficult to find ways to outmaneuver risk adjustment for financial gains. More research is needed to assess payment-specific issues including risk selection for future new risk adjustment models. Equally importantly, from the clinical care perspective, more accurate identification of patients with low and high future health care needs can help care management programs effectively target appropriate patients for interventions.

The study has a few limitations. The distribution of total health care costs is highly skewed with large outliers. We conducted a sensitivity analysis by assigning $134,074 (99^th^ percentile of the distribution of total costs in 2013) to all cases with 2013 health care costs over that amount. The sensitivity analysis results did not alter the directions of our findings although the differences in model performance tended to be less pronounced. Although we used ICD-9-CM diagnosis codes in this study, EDCs derived from ICD-9-CM are consistent with those derived from ICD-10-CM. Thus, our study results are applicable to newer health care data with ICD-10-CM as well. This study did not test all regression techniques. However, we tested a generalized linear model with the log link and gamma distribution, which failed to show consistent advantages over standard linear regression. We also tested several more advanced machine learning techniques including random forests and neural network and found no better overall performance than penalized regression. As the study used administrative claims from a particular large health plan in IMS database, the results may not be generalizable to other health plans or to patients under 65 years old. The sample size of this study was limited. Further research is needed to confirm the findings in larger and more diverse samples and to further establish external validity using test data drawn from a different time period or from a different health plan. We also caution that the clear-cut favorable bias-variance trade-off of penalized regression observed in this study may change with a different outcome variable or even a different data source.

In conclusion, this study demonstrated the advantages of using transparent and easy-to-interpret penalized regression models for predicting future health care costs in older adults relative to standard linear regression. In particular, lasso regression showed better prediction performance across different levels of predicted risk. Such predictive analytic techniques, while incorporating underlying machine learning principles, still embody the familiar linear regression framework and provide transparence and interpretability with a gain in prediction performance. As digital data sources become ever more ubiquitous in the health care sector, it is imperative that advances in data science be considered and embraced as appropriate based on transparent and rigorous assessments. Health care insurers, providers and policy makers may benefit from adopting penalized regression such as lasso regression for cost prediction to improve risk adjustment and population health management and thus better address the underlying needs and risk of the populations they serve.

## Supporting information

S1 Table Proportions of patients with EDCs (n = 277) and RxMGs (n = 67) in 2012 in the training sample. (DOCX)

S2 Table Prediction performance of elastic net regression models, by deciles of predicted costs. (DOCX)
