# Supplementary material for: Exploring the use of machine learning for risk adjustment: A comparison of standard and penalized linear regression models in predicting health care costs in older adults
Source: PLoS One. 2019 Mar 6;14(3):e0213258. doi: 10.1371/journal.pone.0213258 (PMC6402678; doi:10.1371/journal.pone.0213258)
Supplement: S1 Table — (DOCX) [file pone.0213258.s001.docx]

**Online Supplement #1:**

**Proportions of Patients with EDCs (n=277) and RxMGs (n=67) in 2012 in the Training Sample**

| **Comorbidity indicators** | **Comorbidity indicators - labels** | **Prevalence in 2012** | **Top 50 most prevalent comorbidities selected by lasso regression** |
| --- | --- | --- | --- |
| RxMG | Cardiovascular / High Blood Pressure | 58.38% |  |
| EDC | Hypertension, w/o major complications | 52.60% | * |
| RxMG | Cardiovascular / Disorders of Lipid Metabolism | 48.01% |  |
| RxMG | Infections / Acute Minor | 47.82% |  |
| EDC | Disorders of lipid metabolism | 42.76% |  |
| EDC | Preventive care | 38.42% |  |
| EDC | Administrative concerns and non-specific laboratory abnormalities | 36.72% | * |
| RxMG | General Signs and Symptoms / Pain | 29.59% | * |
| EDC | Musculoskeletal signs and symptoms | 29.12% | * |
| RxMG | Gastrointestinal/Hepatic / Peptic Disease | 23.85% | * |
| EDC | Cataract, aphakia | 23.56% | * |
| RxMG | Skin / Acute and Recurrent | 23.03% |  |
| RxMG | Other and Non-Specific Medications | 21.83% | * |
| EDC | Skin keratoses | 21.40% |  |
| EDC | Low back pain | 20.86% | * |
| EDC | Surgical aftercare | 20.45% |  |
| EDC | Benign and unspecified neoplasm | 20.00% | * |
| RxMG | Genito-Urinary / Acute Minor | 18.62% | * |
| RxMG | Psychosocial / Depression | 18.11% | * |
| EDC | Degenerative joint disease | 17.13% | * |
| EDC | Ischemic heart disease (excluding acute myocardial infarction) | 17.00% | * |
| RxMG | General Signs and Symptoms / Pain and Inflammation | 16.36% | * |
| RxMG | Cardiovascular / Chronic Medical | 15.87% | * |
| RxMG | Endocrine / Thyroid Disorders | 15.79% |  |
| EDC | Nonspecific signs and symptoms | 15.57% | * |
| RxMG | Cardiovascular / Vascular Disorders | 15.08% | * |
| RxMG | Musculoskeletal / Inflammatory Conditions | 14.20% | * |
| RxMG | Allergy/Immunology / Chronic Inflammatory | 13.54% | * |
| EDC | Cardiac arrhythmia | 13.31% | * |
| RxMG | Endocrine / Diabetes Without Insulin | 13.28% |  |
| RxMG | Respiratory / Airway Hyperactivity | 12.52% | * |
| EDC | Cardiovascular signs and symptoms | 12.38% |  |
| EDC | Glaucoma | 12.24% | * |
| RxMG | Allergy/Immunology / Acute Minor | 12.23% |  |
| EDC | Gastroesophageal reflux | 12.09% |  |
| RxMG | Psychosocial / Anxiety | 12.06% |  |
| EDC | Respiratory signs and symptoms | 11.95% | * |
| EDC | Musculoskeletal disorders, other | 11.86% | * |
| EDC | Urinary symptoms | 10.95% | * |
| EDC | Bursitis, synovitis, tenosynovitis | 10.89% | * |
| EDC | Hypothyroidism | 10.84% |  |
| EDC | Acute lower respiratory tract infection | 10.51% | * |
| RxMG | Respiratory / Acute Minor | 10.51% | * |
| EDC | Eye, other disorders | 10.24% | * |
| EDC | Dermatitis and eczema | 9.64% |  |
| RxMG | Endocrine / Chronic Medical | 9.32% | * |
| EDC | Peripheral neuropathy, neuritis | 9.29% | * |
| EDC | Dermatophytoses | 9.25% |  |
| EDC | Debility and undue fatigue | 9.14% |  |
| EDC | Chest pain | 9.02% | * |
| EDC | Type 2 diabetes, w/o complication | 8.95% | * |
| EDC | Prostatic hypertrophy | 8.85% | * |
| EDC | Type 2 diabetes, w/ complication | 8.72% | * |
| EDC | Abdominal pain | 8.53% |  |
| EDC | Iron deficiency, other deficiency anemias | 8.45% | * |
| RxMG | Eye / Acute Minor: Curative | 8.45% |  |
| RxMG | Neurologic / Seizure Disorder | 8.41% | * |
| RxMG | Gastrointestinal/Hepatic / Acute Minor | 8.39% |  |
| EDC | Cerebrovascular disease | 8.38% | * |
| EDC | Acute upper respiratory tract infection | 8.35% |  |
| EDC | Sinusitis | 8.29% |  |
| EDC | Benign neoplasm of skin and subcutaneous tissues | 8.21% | * |
| EDC | Allergic rhinitis | 8.18% |  |
| EDC | Cervical pain syndromes | 8.03% | * |
| RxMG | Eye / Acute Minor: Palliative | 7.96% | * |
| EDC | Urinary tract infections | 7.92% | * |
| RxMG | Cardiovascular / Congestive Heart Failure | 7.82% |  |
| EDC | Gastrointestinal signs and symptoms | 7.79% | * |
| EDC | Cough | 7.67% | * |
| EDC | Other skin disorders | 7.58% | * |
| EDC | Retinal disorders (excluding diabetic retinopathy) | 7.39% | * |
| EDC | Disorders of the eyelid and lacrimal duct | 7.36% |  |
| EDC | Acute sprains and strains | 7.17% | * |
| EDC | Emphysema, chronic bronchitis, COPD | 7.13% | * |
| EDC | Malignant neoplasms of the skin | 7.05% | * |
| EDC | Neurologic signs and symptoms | 6.93% | * |
| RxMG | Endocrine / Bone Disorders | 6.90% |  |
| RxMG | Eye / Glaucoma | 6.85% |  |
| EDC | Age-related macular degeneration | 6.74% |  |
| RxMG | Psychosocial / Sleep disorders | 6.55% |  |
| RxMG | General Signs and Symptoms / Nausea and Vomiting | 6.42% |  |
| EDC | Nonfungal infections of skin and subcutaneous tissue | 6.35% |  |
| EDC | Hypertension, with major complications | 6.32% |  |
| EDC | Vertiginous syndromes | 6.14% |  |
| EDC | Other endocrine disorders | 6.12% |  |
| EDC | Generalized atherosclerosis | 6.07% |  |
| EDC | Diverticular disease of colon | 5.82% |  |
| EDC | Fluid/electrolyte disturbances | 5.68% |  |
| EDC | Contusions and abrasions | 5.63% |  |
| EDC | Wax in ear | 5.63% |  |
| EDC | Cardiac valve disorders | 5.50% |  |
| EDC | Nutritional deficiencies | 5.49% |  |
| EDC | Edema | 5.45% |  |
| EDC | Neurologic disorders, other | 5.38% |  |
| EDC | Deafness, hearing loss | 5.32% |  |
| EDC | Ophthalmic signs and symptoms | 5.30% |  |
| EDC | Chronic renal failure | 5.26% |  |
| EDC | Osteoporosis | 5.14% |  |
| EDC | Conjunctivitis, keratitis | 4.82% |  |
| EDC | Gastroenteritis | 4.57% |  |
| EDC | Acquired foot deformities | 4.50% |  |
| EDC | Sleep apnea | 4.42% |  |
| EDC | Congestive heart failure | 4.41% |  |
| EDC | Depression | 4.36% |  |
| EDC | Asthma, w/o status asthmaticus | 4.33% |  |
| EDC | Arthropathy | 4.27% |  |
| EDC | Diseases of nail | 4.22% |  |
| EDC | Anorectal conditions | 4.19% |  |
| EDC | Peripheral vascular disease | 4.19% |  |
| RxMG | Musculoskeletal / Gout | 4.11% |  |
| EDC | Genito-urinary disorders, other | 4.07% |  |
| EDC | Anxiety, neuroses | 4.06% |  |
| RxMG | Respiratory / Chronic Medical | 3.96% |  |
| EDC | Refractive errors | 3.96% |  |
| EDC | Lacerations | 3.93% |  |
| EDC | Malignant neoplasms, prostate | 3.91% |  |
| RxMG | Endocrine / Diabetes With Insulin | 3.82% |  |
| EDC | Fractures (excluding digits) | 3.73% |  |
| EDC | Peptic ulcer disease | 3.72% |  |
| EDC | Cardiovascular disorders, other | 3.57% |  |
| RxMG | Malignancies | 3.56% |  |
| EDC | Other breast disorders | 3.52% |  |
| EDC | Joint disorders, trauma related | 3.52% |  |
| EDC | Sleep problems | 3.52% |  |
| EDC | Exanthems | 3.48% |  |
| EDC | Dementia | 3.45% |  |
| EDC | Other male genital disease | 3.39% |  |
| RxMG | General Signs and Symptoms / Severe Pain | 3.37% |  |
| EDC | Otitis media | 3.36% |  |
| EDC | Nausea, vomiting | 3.36% |  |
| RxMG | Neurologic / Alzheimers Disease | 3.35% |  |
| EDC | Respiratory disorders, other | 3.27% |  |
| EDC | Constipation | 3.26% |  |
| EDC | Headaches | 3.05% |  |
| EDC | Syncope | 3.02% |  |
| EDC | Dermatologic signs and symptoms | 2.96% |  |
| EDC | Incontinence | 2.86% |  |
| EDC | Infections of eyelid | 2.69% |  |
| EDC | Obesity | 2.67% |  |
| RxMG | Infections / Severe Acute Major | 2.66% |  |
| EDC | ENT disorders, other | 2.64% |  |
| EDC | Renal disorders, other | 2.53% |  |
| EDC | Gout | 2.42% |  |
| RxMG | Neurologic / Parkinsons Disease | 2.32% |  |
| EDC | Gastrointestinal/hepatic disorders, other | 2.32% |  |
| EDC | Sebaceous cyst | 2.29% |  |
| EDC | Vaginitis, vulvitis, cervicitis | 2.24% |  |
| EDC | Malignant neoplasms, breast | 2.21% |  |
| EDC | Chronic ulcer of the skin | 2.16% |  |
| EDC | Hematologic disorders, other | 2.04% |  |
| EDC | Cardiomyopathy | 2.03% |  |
| EDC | Renal calculi | 1.99% |  |
| EDC | Other inflammatory conditions of skin | 1.96% |  |
| EDC | Fever | 1.90% |  |
| EDC | Gastrointestinal obstruction/perforation | 1.86% |  |
| EDC | Major depression | 1.83% |  |
| EDC | Autoimmune and connective tissue diseases | 1.83% |  |
| EDC | Aortic aneurysm | 1.81% |  |
| EDC | Menopausal symptoms | 1.78% |  |
| EDC | External abdominal hernias, hydroceles | 1.76% |  |
| EDC | Acute renal failure | 1.75% |  |
| RxMG | Ears, Nose, Throat / Acute Minor | 1.73% |  |
| EDC | Low impact malignant neoplasms | 1.69% |  |
| EDC | Complications of mechanical devices | 1.63% |  |
| EDC | Acne | 1.60% |  |
| RxMG | Psychosocial / Chronic Unstable | 1.58% |  |
| EDC | Diabetic retinopathy | 1.57% |  |
| EDC | Rheumatoid arthritis | 1.54% |  |
| EDC | Deep vein thrombosis | 1.50% |  |
| EDC | Varicose veins of lower extremities | 1.49% |  |
| EDC | Viral warts and molluscum contagiosum | 1.46% |  |
| EDC | Adjustment disorder | 1.46% |  |
| EDC | Tobacco use | 1.44% |  |
| EDC | Infections, other | 1.43% |  |
| EDC | Chronic respiratory failure | 1.41% |  |
| EDC | Disorders of sebaceous glands | 1.40% |  |
| EDC | Inherited metabolic disorders | 1.40% |  |
| EDC | Adverse events from medical/surgical procedures | 1.37% |  |
| EDC | Head injury | 1.37% |  |
| EDC | Type 1 diabetes, w/ complication | 1.37% |  |
| EDC | Irritable bowel syndrome | 1.32% |  |
| EDC | Allergic reactions | 1.27% |  |
| EDC | Otitis externa | 1.24% |  |
| EDC | Cholelithiasis, cholecystitis | 1.21% |  |
| EDC | Disorders of mouth | 1.20% |  |
| EDC | High impact malignant neoplasms | 1.19% |  |
| EDC | Utero-vaginal prolapse | 1.16% |  |
| EDC | Chronic cystic disease of the breast | 1.12% |  |
| EDC | Parkinsons disease | 1.11% |  |
| EDC | Tinnitus | 1.07% |  |
| RxMG | Psychosocial / Bipolar disorder | 1.04% |  |
| EDC | Chronic liver disease | 1.04% |  |
| EDC | Seizure disorder | 1.02% |  |
| EDC | Migraines | 1.01% |  |
| EDC | Viral syndromes | 1.00% |  |
| EDC | Acute respiratory failure | 1.00% |  |
| EDC | Fungal infections | 1.00% |  |
| EDC | Psoriasis | 0.97% |  |
| EDC | Nutritional disorders, other | 0.96% |  |
| EDC | Kyphoscoliosis | 0.94% |  |
| EDC | Heart murmur | 0.92% |  |
| EDC | Adverse effects of medicinal agents | 0.90% |  |
| EDC | Acute myocardial infarction | 0.89% |  |
| EDC | Disorders of the immune system | 0.88% |  |
| RxMG | Gastrointestinal/Hepatic / Inflammatory Bowel Disease | 0.88% |  |
| EDC | Aspiration and bacterial pneumonias | 0.87% |  |
| EDC | Disease of hair and hair follicles | 0.85% |  |
| RxMG | Skin / Acne | 0.84% |  |
| EDC | Epistaxis | 0.83% |  |
| EDC | Prostatitis | 0.82% |  |
| EDC | Malignant neoplasms, bladder | 0.82% |  |
| EDC | Septicemia | 0.80% |  |
| EDC | Malignant neoplasms, colorectal | 0.79% |  |
| EDC | Deviated nasal septum | 0.78% |  |
| EDC | Female genital symptoms | 0.77% |  |
| EDC | Strabismus, amblyopia | 0.72% |  |
| EDC | Schizophrenia and affective psychosis | 0.71% |  |
| EDC | Malignant neoplasms, lymphomas | 0.70% |  |
| EDC | Inflammatory bowel disease | 0.70% |  |
| EDC | Hemophilia, coagulation disorder | 0.68% |  |
| EDC | Lymphadenopathy | 0.67% |  |
| RxMG | Neurologic / Migraine Headache | 0.67% |  |
| EDC | Traumatic injuries of eye | 0.57% |  |
| EDC | Organic brain syndrome | 0.56% |  |
| EDC | Substance use | 0.56% |  |
| EDC | Pulmonary embolism | 0.55% |  |
| EDC | Paralytic syndromes, other | 0.53% |  |
| EDC | Congenital heart disease | 0.53% |  |
| EDC | Fracture of neck of femur (hip) | 0.53% |  |
| EDC | Malignant neoplasms, lung | 0.50% |  |
| RxMG | Psychosocial / Attention Deficit Hyperactivity Disorder | 0.48% |  |
| RxMG | Infections / Acute Major | 0.48% |  |
| EDC | Thrombophlebitis | 0.46% |  |
| EDC | Female gynecologic conditions, other | 0.46% |  |
| RxMG | Neurologic / Chronic Medical | 0.45% |  |
| RxMG | Genito-Urinary / Chronic Renal Failure | 0.44% |  |
| EDC | Asthma, with status asthmaticus | 0.43% |  |
| EDC | Sexually transmitted diseases | 0.43% |  |
| EDC | Menstrual disorders | 0.42% |  |
| EDC | Pigmented nevus | 0.40% |  |
| EDC | Bipolar disorder | 0.40% |  |
| EDC | Sleep disorders of nonorganic origin | 0.40% |  |
| EDC | Fractures and dislocations/digits only | 0.39% |  |
| EDC | Stricture of urethra | 0.39% |  |
| EDC | Blindness | 0.39% |  |
| RxMG | Female Reproductive / Hormone Regulation | 0.39% |  |
| EDC | Spinal cord injury/disorders | 0.38% |  |
| EDC | ESRD | 0.36% |  |
| EDC | Malignant neoplasms, kidney | 0.35% |  |
| EDC | Psychosexual | 0.33% |  |
| EDC | Developmental disorder | 0.33% |  |
| RxMG | Skin / Chronic Medical | 0.33% |  |
| EDC | Congenital anomalies of limbs, hands, and feet | 0.32% |  |
| EDC | Foreign body in eye | 0.32% |  |
| EDC | Type 1 diabetes, w/o complication | 0.32% |  |
| EDC | Alimentary or excretory surgical openings | 0.31% |  |
| RxMG | Allergy/Immunology / Transplant | 0.30% |  |
| EDC | Chronic pancreatitis | 0.30% |  |
| EDC | Toxic effects of nonmedicinal agents | 0.29% |  |
| EDC | Acute pancreatitis | 0.28% |  |
| EDC | Transplant status | 0.26% |  |
| EDC | Nephritis, nephrosis | 0.26% |  |
| EDC | Malignant neoplasms, cervix, uterus | 0.26% |  |
| EDC | Delirium | 0.26% |  |
| EDC | Psych-physiologic and somatoform disorders | 0.24% |  |
| RxMG | Psychosocial / Tobacco Cessation | 0.24% |  |
| EDC | Failure to thrive | 0.24% |  |
| EDC | Foreign body in ears, nose, or throat | 0.23% |  |
| EDC | Keloid | 0.23% |  |
| EDC | Disorders of teeth | 0.23% |  |
| EDC | Temporomandibular joint disease | 0.22% |  |
| EDC | Lactose intolerance | 0.21% |  |
| EDC | Abnormal pap smear | 0.21% |  |
| EDC | Stomatitis | 0.19% |  |
| EDC | Ovarian cyst | 0.19% |  |
| EDC | Central nervous system infections | 0.19% |  |
| RxMG | Gastrointestinal/Hepatic / Pancreatic Disorder | 0.19% |  |
| EDC | Multiple sclerosis | 0.18% |  |
| EDC | Acute hepatitis | 0.18% |  |
| EDC | Raynauds syndrome | 0.18% |  |
| EDC | Burns--2nd and 3rd degree | 0.18% |  |
| EDC | Amputation status | 0.18% |  |
| EDC | Attention deficit disorder | 0.17% |  |
| RxMG | Infections / HIV/AIDS | 0.16% |  |
| EDC | Muscular dystrophy | 0.16% |  |
| EDC | Cardiac arrest, shock | 0.16% |  |
| RxMG | Allergy/Immunology / Immune Disorders | 0.16% |  |
| EDC | Malignant neoplasms, ovary | 0.16% |  |
| RxMG | Psychosocial / Addiction | 0.15% |  |
| EDC | Impetigo | 0.15% |  |
| RxMG | Gastrointestinal/Hepatic / Chronic Liver Disease | 0.14% |  |
| EDC | Family and social problems | 0.13% |  |
| RxMG | Gastrointestinal/Hepatic / Chronic Stable | 0.12% |  |
| EDC | Chronic pharyngitis and tonsillitis | 0.11% |  |
| EDC | Other hemolytic anemias | 0.11% |  |
| EDC | Torticollis | 0.11% |  |
| RxMG | Infections / Tuberculosis | 0.10% |  |
| EDC | Lyme disease | 0.10% |  |
| EDC | Appendicitis | 0.09% |  |
| EDC | Malignant neoplasms, liver and biliary tract | 0.09% |  |
| EDC | Quadriplegia and paraplegia | 0.08% |  |
| EDC | HIV, AIDS | 0.07% |  |
| EDC | Malignant neoplasms, esophagus | 0.07% |  |
| EDC | Gingivitis | 0.07% |  |
| EDC | Tracheostomy | 0.07% |  |
| EDC | Personality disorders | 0.07% |  |
| EDC | Malignant neoplasms, stomach | 0.07% |  |
| RxMG | Respiratory / Cystic Fibrosis | 0.06% |  |
| RxMG | Toxic Effects/Adverse Effects / Acute Major | 0.06% |  |
| EDC | Scabies and pediculosis | 0.06% |  |
| EDC | Malignant neoplasms, pancreas | 0.06% |  |
| EDC | Psychological disorders of childhood | 0.05% |  |
| EDC | Vesicoureteral reflux | 0.05% |  |
| EDC | Acute leukemia | 0.05% |  |
| EDC | Aplastic anemia | 0.05% |  |
| RxMG | Hematologic / Coagulation Disorders | 0.04% |  |
| RxMG | Endocrine / Weight Control | 0.04% |  |
| RxMG | Female Reproductive / Pregnancy and Delivery | 0.03% |  |
| EDC | Psychologic signs and symptoms | 0.03% |  |
| EDC | Tuberculosis infection | 0.03% |  |
| EDC | Burns--1st degree | 0.03% |  |
| EDC | Endometriosis | 0.03% |  |
| EDC | Pregnancy and delivery with complications | 0.03% |  |
| EDC | Disorders of newborn period | 0.02% |  |
| EDC | Eating disorder | 0.02% |  |
| EDC | Hypospadias, other penile anomalies | 0.02% |  |
| EDC | Pregnancy and delivery, uncomplicated | 0.01% |  |
| EDC | Female infertility | 0.01% |  |
| EDC | Cystic fibrosis | 0.01% |  |
| EDC | Cerebral palsy | 0.01% |  |
| EDC | Impulse control | 0.01% |  |
| EDC | Chromosomal anomalies | 0.01% |  |
| RxMG | Endocrine / Growth Problems | 0.01% |  |
| EDC | Sickle cell disease | 0.01% |  |
| EDC | Undescended testes | 0.01% |  |
| EDC | Congenital hip dislocation | 0.01% |  |
| EDC | Contraception | 0.00% |  |
| EDC | Newborn status, complicated | 0.00% |  |
| RxMG | Psychosocial / Acute Minor | 0.00% |  |
| EDC | Cleft lip and palate | 0.00% |  |
| EDC | Newborn status, uncomplicated | 0.00% |  |
| EDC | Infectious mononucleosis | 0.00% |  |
| RxMG | Female Reproductive / Infertility | 0.00% |  |
| EDC | Neonatal jaundice | 0.00% |  |

EDC: Expanded Diagnosis Clusters; RxMG: Rx-defined Morbidity Groups

* indicates the top 50 most prevalent comorbdity indicators selected by lasso regression as important variables. A total of 173 comorbdity indicators were selected by lasso regression as important variables.
