# Supplementary material for: Exploring the use of machine learning for risk adjustment: A comparison of standard and penalized linear regression models in predicting health care costs in older adults
Source: PLoS One. 2019 Mar 6;14(3):e0213258. doi: 10.1371/journal.pone.0213258 (PMC6402678; doi:10.1371/journal.pone.0213258)
Supplement: S2 Table — (DOCX) [file pone.0213258.s002.docx]

**Online Supplement #2:**

**Table A: Prediction Performance of Elastic Net Regression Models using 2012 Predictors, by Deciles of Predicted Costs**

| **Decile n** | | **Mean predicted costs ($)** | **Mean actual costs ($)** | **RMSE ($)** | **MAPE ($)** | **PR** |
| --- | --- | --- | --- | --- | --- | --- |
| elastic net alpha= 0.1 | | | | | | |
| 1 | 2,037 | 3,511 | 3,551 | 15,108 | 4,886 | 0.989 |
| 2 | 2,037 | 5,446 | 5,202 | 18,995 | 6,458 | 1.047 |
| 3 | 2,037 | 7,218 | 6,964 | 17,399 | 7,504 | 1.036 |
| 4 | 2,037 | 9,105 | 8,900 | 23,540 | 8,994 | 1.023 |
| 5 | 2,037 | 11,146 | 11,426 | 22,734 | 10,999 | 0.976 |
| 6 | 2,037 | 13,584 | 14,121 | 28,786 | 13,690 | 0.962 |
| 7 | 2,037 | 16,547 | 17,441 | 31,287 | 16,219 | 0.949 |
| 8 | 2,037 | 20,426 | 19,682 | 30,795 | 17,609 | 1.038 |
| 9 | 2,037 | 26,689 | 26,437 | 39,936 | 23,099 | 1.010 |
| 10 | 2,036 | 49,710 | 49,129 | 80,075 | 43,000 | 1.012 |
| elastic net alpha= 0.5 | | | | | | |
| 1 | 2,037 | 3,519 | 3,577 | 15,113 | 4,898 | 0.984 |
| 2 | 2,037 | 5,449 | 5,150 | 18,739 | 6,395 | 1.058 |
| 3 | 2,037 | 7,211 | 6,934 | 17,350 | 7,485 | 1.040 |
| 4 | 2,037 | 9,091 | 8,948 | 23,627 | 9,002 | 1.016 |
| 5 | 2,037 | 11,132 | 11,641 | 23,482 | 11,195 | 0.956 |
| 6 | 2,037 | 13,570 | 14,037 | 28,480 | 13,553 | 0.967 |
| 7 | 2,037 | 16,528 | 17,550 | 32,062 | 16,405 | 0.942 |
| 8 | 2,037 | 20,396 | 19,578 | 30,001 | 17,496 | 1.042 |
| 9 | 2,037 | 26,637 | 26,503 | 39,962 | 23,068 | 1.005 |
| 10 | 2,036 | 49,845 | 48,935 | 80,074 | 43,004 | 1.019 |
| elastic net alpha= 0.9 | | | | | | |
| 1 | 2,037 | 3,505 | 3,580 | 15,115 | 4,894 | 0.979 |
| 2 | 2,037 | 5,441 | 5,096 | 18,625 | 6,340 | 1.068 |
| 3 | 2,037 | 7,203 | 6,980 | 17,472 | 7,527 | 1.032 |
| 4 | 2,037 | 9,084 | 8,946 | 23,623 | 8,987 | 1.015 |
| 5 | 2,037 | 11,126 | 11,675 | 23,490 | 11,207 | 0.953 |
| 6 | 2,037 | 13,566 | 14,025 | 28,479 | 13,547 | 0.967 |
| 7 | 2,037 | 16,525 | 17,580 | 32,182 | 16,470 | 0.940 |
| 8 | 2,037 | 20,395 | 19,526 | 29,872 | 17,437 | 1.045 |
| 9 | 2,037 | 26,640 | 26,473 | 39,955 | 23,057 | 1.006 |
| 10 | 2,036 | 49,888 | 48,971 | 80,088 | 43,034 | 1.019 |

RMSE: root mean squared error; MAPE: mean absolute prediction error; PR: prediction ratio

**Table B: Prediction Performance of Elastic Net Regression Models using 2009-2012 Predictors, by Deciles of Predicted Costs**

| **Decile n** | | **Mean predicted costs ($)** | **Mean actual costs ($)** | **RMSE ($)** | **MAPE ($)** | **PR** |
| --- | --- | --- | --- | --- | --- | --- |
| elastic net alpha= 0.1 | | | | | | |
| 1 | 2,037 | 3,606 | 3,137 | 12,705 | 4,564 | 1.150 |
| 2 | 2,037 | 5,502 | 5,417 | 19,395 | 6,529 | 1.016 |
| 3 | 2,037 | 7,263 | 7,228 | 21,681 | 7,726 | 1.005 |
| 4 | 2,037 | 9,144 | 9,225 | 21,031 | 9,344 | 0.991 |
| 5 | 2,037 | 11,234 | 11,237 | 23,886 | 10,945 | 1.000 |
| 6 | 2,037 | 13,650 | 13,995 | 26,913 | 13,460 | 0.975 |
| 7 | 2,037 | 16,544 | 17,049 | 31,045 | 16,002 | 0.970 |
| 8 | 2,037 | 20,524 | 20,230 | 33,789 | 18,121 | 1.015 |
| 9 | 2,037 | 26,724 | 25,616 | 36,599 | 22,451 | 1.043 |
| 10 | 2,036 | 49,334 | 49,718 | 79,659 | 42,957 | 0.992 |
| elastic net alpha= 0.5 | | | | | | |
| 1 | 2,037 | 3,510 | 3,153 | 12,715 | 4,500 | 1.113 |
| 2 | 2,037 | 5,416 | 5,421 | 19,286 | 6,440 | 0.999 |
| 3 | 2,037 | 7,186 | 7,117 | 21,431 | 7,614 | 1.010 |
| 4 | 2,037 | 9,075 | 9,401 | 21,541 | 9,494 | 0.965 |
| 5 | 2,037 | 11,182 | 11,131 | 23,819 | 10,767 | 1.005 |
| 6 | 2,037 | 13,602 | 14,463 | 28,479 | 13,819 | 0.940 |
| 7 | 2,037 | 16,514 | 16,902 | 30,053 | 15,889 | 0.977 |
| 8 | 2,037 | 20,516 | 20,085 | 33,674 | 17,986 | 1.021 |
| 9 | 2,037 | 26,745 | 25,560 | 37,084 | 22,507 | 1.046 |
| 10 | 2,036 | 49,737 | 49,619 | 79,437 | 43,073 | 1.002 |
| elastic net alpha= 0.9 | | | | | | |
| 1 | 2,037 | 3,497 | 3,154 | 12,716 | 4,493 | 1.109 |
| 2 | 2,037 | 5,406 | 5,419 | 19,286 | 6,434 | 0.998 |
| 3 | 2,037 | 7,177 | 7,116 | 21,429 | 7,603 | 1.009 |
| 4 | 2,037 | 9,067 | 9,363 | 21,522 | 9,476 | 0.968 |
| 5 | 2,037 | 11,176 | 11,092 | 23,787 | 10,753 | 1.008 |
| 6 | 2,037 | 13,597 | 14,544 | 28,509 | 13,837 | 0.935 |
| 7 | 2,037 | 16,511 | 16,995 | 30,173 | 15,943 | 0.972 |
| 8 | 2,037 | 20,516 | 19,906 | 33,418 | 17,860 | 1.031 |
| 9 | 2,037 | 26,748 | 25,628 | 37,237 | 22,592 | 1.044 |
| 10 | 2,036 | 49,784 | 49,635 | 79,454 | 43,098 | 1.003 |

RMSE: root mean squared error; MAPE: mean absolute prediction error; PR: prediction ratio
